# A survey of epibiont hydrozoans on *Sargassum*

**DOI:** 10.7717/peerj.15423

**Published:** 2023-05-30

**Authors:** Cecilia Odette Carral-Murrieta, Antonio C. Marques, Elisa Serviere-Zaragoza, Mariae C. Estrada-González, Amanda F. Cunha, Marina O. Fernandez, Alejandra Mazariegos-Villarreal, Karla León-Cisneros, Juan López-Vivas, José Agüero, María A. Mendoza-Becerril

**Affiliations:** 1 Centro de Investigaciones Biológicas del Noroeste (CIBNOR), La Paz, Baja California Sur, Mexico; 2 Departamento de Zoologia, Universidade de São Paulo, São Paulo, São Paulo, Brazil; 3 Medusozoa México, La Paz, Baja California Sur, Mexico; 4 Departamento de Biologia Animal, Centro de Ciências Biológicas e da Saúde, Universidade Federal de Viçosa, Viçosa, Minas Gerais, Brazil; 5 Departamento Académico de Ciencias Marinas y Costeras, Universidad Autónoma de Baja California Sur, La Paz, Baja California Sur, Mexico; 6 El Colegio de la Frontera Sur (ECOSUR), Chetumal, Quintana Roo, Mexico

**Keywords:** Epibionts, Hydrozoa, Macroalgae, Medusozoa, Polyps

## Abstract

The brown alga *Sargassum* provides a natural substrate occupied by hydrozoans in shallow marine waters. A global count in 2007 listed 39 epibiotic species of Hydrozoa growing on *Sargassum*, but more studies have been published since, therefore, an update is timely, particularly due to the increased abundance of *Sargassum* in the Caribbean. This review, based on a recent literature survey and new records from Mexico, includes 133 publications of epibiotic hydrozoans on *Sargassum* spanning 220 years, from 1802 to 2022. A total of 131 hydrozoan species were recorded on 26 species of *Sargassum*, most belonging to the subclass Hydroidolina (130), with only one record of a trachyline medusa (*Gonionemus vertens*, subclass Trachylinae). Most publications centered on the Tropical Atlantic, where the greatest number of hydrozoan species (67 species) were recorded. All hydrozoan species possess a hydrorhiza, except one hydromedusae species that attach to *Sargassum* via adhesive tentacles. Most of the hydrozoan species associated with *Sargassum* exhibited a benthic life cycle (93 species) and are comprised of erect, branched colonies (67 species) and large hydrothecae (69 species). Although the number of studies of epibiotic hydrozoans on *Sargassum* has increased since the mid-20th century, nevertheless hydrozoan richness has not reached an asymptote. Therefore, more sampling of *Sargassum* species would likely identify more hydrozoan species associated with *Sargassum*, especially among benthic *Sargassum*, and might help reveal potential biogeographical and ecological patterns between *Sargassum* and hydrozoan epibionts.

## Introduction

Hydrozoa is a class of Cnidaria with diverse and complex life cycles, characterized by an alternation between a benthic polyp stage (hydroid) and a planktonic medusa stage (hydromedusae). One of these stages can be reduced or absent in several lineages (Marques & Collins, 2004; Cartwright & Nawrocki, 2010). Marine hydroids contribute to the complexity of benthic habitats by providing refuge for many species and prey for predators (Di Camillo et al., 2017). Hydrozoans are epibionts of plants and animals and basibionts (host of epibionts) of viruses, bacteria, and protists (Brinckmann, 1964; Fraser, Capa & Schuchert, 2006; McGaw, 2006; Bavestrello et al., 2008; Stabili et al., 2008; Daley, Urban-Rich & Moisander, 2016; Di Camillo et al., 2017; Gravili, Cozzoli & Gambi, 2021). Hydroids attach to natural or artificial substrates with their stolons or hydrorhiza, often forming networks or mats (Llobet, Gili & Barangé, 1986; Calder, 1991). A few hydromedusae, such as *Gonionemus vertens* A. Agassiz, 1862, also attach to substrates with adherent tentacles (Tambs-Lyche, 1964).

Hydroids tend to be predominantly substrate generalists (Calder, 1976; Gili, Murillo & Ros, 1989; Mendoza-Becerril, Simões & Genzano, 2018). However, marine macroalgae are common substrates occupied by hydrozoans (hydroids and hydromedusae) in shallow waters (*i.e.,* Nishihira, 1968; Oliveira & Marques, 2007; Mendoza-Becerril, Simões & Genzano, 2018), including red and brown algae, especially species belonging to the genera *Galaxaura* and *Sargassum*, respectively, which have the highest diversity index of epibiotic hydrozoans (Cunha & Jacobucci, 2010; Oliveira & Marques, 2011).

Hydrozoans growing on *Sargassum* have been reported in taxonomic or faunistic studies since the early nineteenth century (Bosc, 1802). Later, a global count listed 39 epibiotic hydrozoans growing on *Sargassum* (Oliveira & Marques, 2007). Subsequently, other studies have been published that include benthic and holopelagic *Sargassum* (*e.g.*, Cunha & Jacobucci, 2010; Galea & Ferry, 2015; Mendoza-Becerril, Simões & Genzano, 2018). The study of *Sargassum* as a basibiont could help to define a tangible scope of work where data can be processed, and biological patterns unraveled similar to other epibionts of these algae (see Carvalho et al., 2022). In addition, studying *Sargassum* as a basibiont can help in making decisions in the face of the ecological, social, and economic impact of *Sargassum* (Joniver et al., 2021; López Miranda et al., 2021) and hydrozoans (Wahl, 2008; Marques, Haddad Jr & Migotto, 2002).

Therefore, this study compiles data of the hydrozoan species reported on macroalgae of the genus *Sargassum* to describe the historical development of hydrozoan epibiont studies. Similarly, we contributed knowledge about the relation between the type, level, and geographic area of epibiont hydrozoans and *Sargassum* diversity or between the morphology of the epibiont hydrozoans and their biodiversity, among others. With this approach, we hope to contribute with a greater understanding of dynamic interactions among these habitat-forming species, as well as to identify potential biogeographical patterns and their relationship with biological and ecological aspects of hydrozoans as epibionts of *Sargassum* worldwide.

## Material and Methods

In this study, we searched for publications listed in Google Scholar (https://scholar.google.com/) and Web of Science (WoS) (https://login.webofknowledge.com) databases published until 2022, using the search terms: “Hydrozoa and *Sargassum*”. We found 867 publications from Google Scholar and 11 from WoS. We excluded duplicate literature results and only reviewed scientific articles that included records of hydrozoans growing on *Sargassum*, resulting in 133 total publications. Additional records from our field samples of *Sargassum horridum* Setchell & N.L. Gardner 1924 and *S. lapazeanum* Setchell & N.L. Gardner 1924 collected manually in La Paz Bay, Baja California Sur (BCS), Mexico, and preserved in ethanol 96%; more details about field samples were included in Table S1. All collected thalli were 7–100 cm in length. *Sargassum* species were morphologically identified according to Andrade-Sorcia, Riosmena-Rodriguez & Muñiz Salazar (2014).

We then entered each study into a matrix that incorporated data from the original publications, including information on the species’ geographic location (locality, country, ocean, and realm), the method used for sampling *Sargassum*, and environmental variables (temperature, salinity, and depth). The classification and taxonomic status of each species were verified based on Maronna et al. (2016) and WoRMS (2022) for hydrozoans and Guiry & Guiry (2022) for *Sargassum*.

Hydrozoan and *Sargassum* species traits were obtained from the articles and complemented by specialized literature (*e.g.*, Parr, 1939; Calder, 1988; Schuchert, 2007). Hydrozoan traits included the type of life cycle (benthic = only polyp stage; meroplanktonic = polyp and medusa stages or polyp and medusoid stages), colony morphology (stolonal; erect and unbranched; erect and branched), and hydrotheca (absent; present or present as pseudohydrotheca; large or small). *Sargassum* traits included the habit (benthic; drifting = *Sargassum* records not identified to the species level and found floating in the water, therefore carried slowly by the currents; holopelagic) and the biogeographic realm (Temperate Northern Pacific; Tropical Eastern Pacific; Temperate South America; Tropical Atlantic; Temperate Northern Atlantic; Western Indo-Pacific; Central Indo-Pacific; Temperate Australasia; widespread). The marine biogeographic realms followed those proposed by Spalding et al. (2007), a global system for coastal and shelf areas that can be cross-referenced to many regional biogeographic classifications. The data matrix with all the information collected is available in Carral-Murrieta et al. (2023).

We plotted both the number of publications per year and the number of publications accumulated over time. The number of publications per marine realm was plotted on a map generated in Quantum GIS (QGIS) software version 3.16.16 (Open Source Geospatial Foundation, Boston, USA).

Our analyses were performed in R packages (R Core Team, 2021) and included only records of *Sargassum* and hydrozoans identified to species. We interpolated and extrapolated curves of estimated species richness per year to compare the species richness of hydrozoans and years with studies that have data on epibiotic hydrozoans on *Sargassum*. We then calculated species richness estimates for twice the number of observed years using the ‘iNEXT’ function of the ‘iNEXT’ package (Chao et al., 2014; Hsieh, Ma & Chao, 2020). We also estimated the species richness asymptote for each year using the Chao method (Chao, 1984; Chao, 1987) with the ‘ChaoRichness’ function of the ‘iNEXT’ package, where the bootstrap standard error and confidence interval were calculated from the logical variable ‘se’ (Chao et al., 2014; Hsieh, Ma & Chao, 2020). We performed the same analyses to compare the species richness of hydrozoans and *Sargassum*, where we interpolated and extrapolated curves of estimated species richness per site for each *Sargassum* species with ten or more records of hydrozoan species. We also compared hydrozoan richness only between marine realms with ten or more hydrozoan records.

A bipartite network of circular visualization was represented in a chord diagram using the ‘circlize’ package (Gu et al., 2014). We made a pie chart to illustrate the numerical proportion of epibiotic hydrozoans reported relative to the type of *Sargassum* species habit: benthic, holopelagic, and undefined (for any *Sargassum* record reported only at the genus level, habitat could not be determined). We also estimated the proportion of hydrozoan records relative to each trait for each *Sargassum* species (life cycle, morphology, and hydrotheca).

We compared the compositions of hydrozoans by *Sargassum* traits using a non-metric multidimensional scaling (NMDS) ordination with 100 random starts using the function ‘metaMDS’ of the ‘vegan’ package (Oksanen et al., 2017). We calculated distances between *Sargassum* species using Sorensen dissimilarities based on presence/absence data. However, we added a dummy hydrozoan species to every *Sargassum* species before calculating dissimilarities because complete dissimilarity between *Sargassum* species with no hydrozoans in common would lead to a loss of information in the analysis (Clarke, Somerfield & Chapman, 2006; Smith, 2017).

We investigated differences in species composition of hydrozoans relative to habitat and distribution of *Sargassum* species using PERMANOVA with 999 permutations. Differences were calculated using a Bray-Curtis distance matrix based on hydrozoan presence/absence on *Sargassum*. Our analyses were performed with the ‘adonis’ function in the ‘vegan’ package (0.4 version). When the *p*-values obtained with PERMANOVA were significant (*p* <0.05), we applied a Bonferroni correction to pairwise tests to avoid false significant results. Pairwise tests were performed with the function ‘pairwiseAdonis’ (Martinez Arbizu, 2020).

## Results

**Figure 1:**
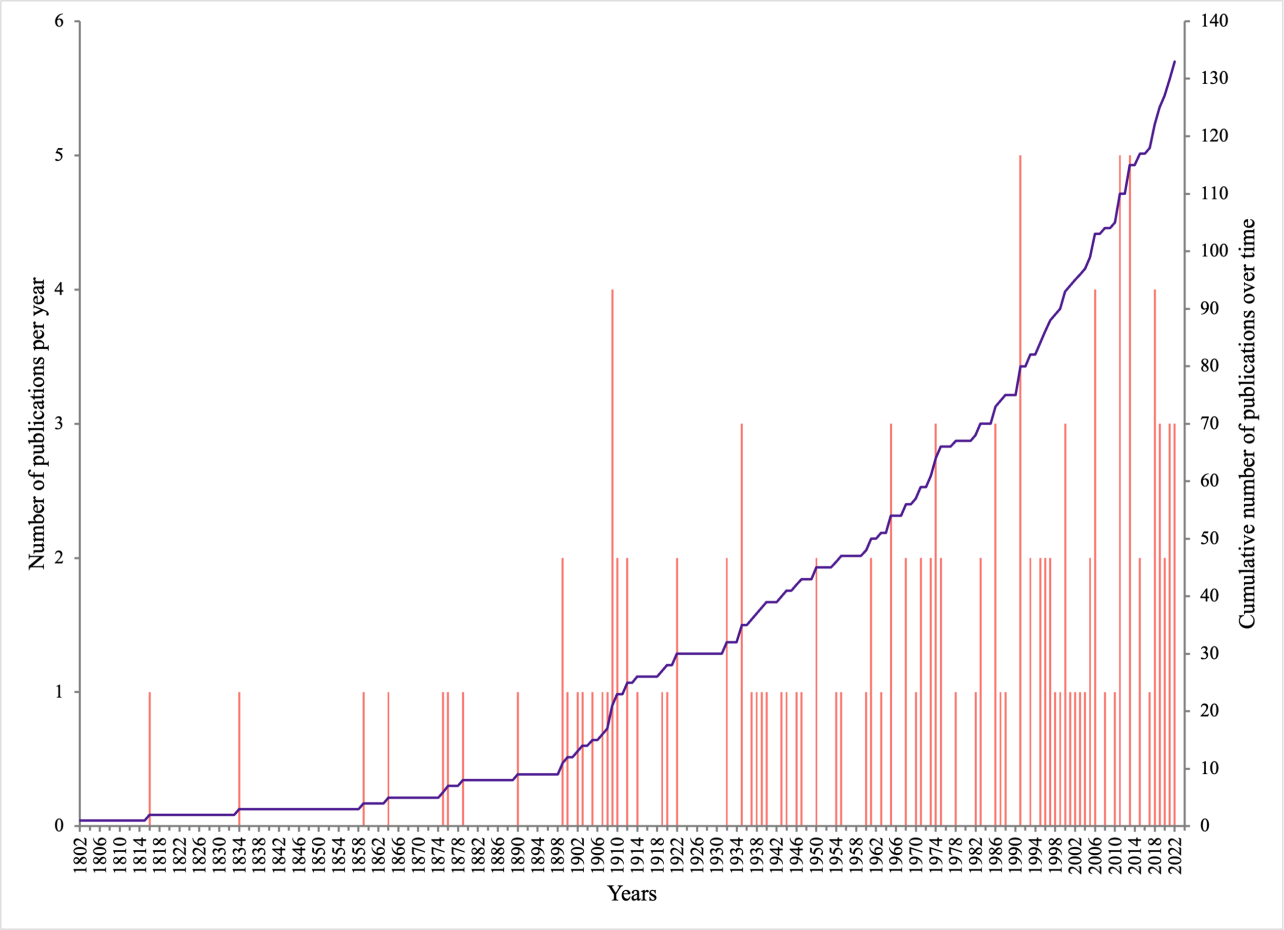
Number of publications with records of epibiotic hydrozoans on *Sargassum*, per year (pink bars), and cumulative number of publications through years 1802 to 2022 (purple line).

**Figure 2:**
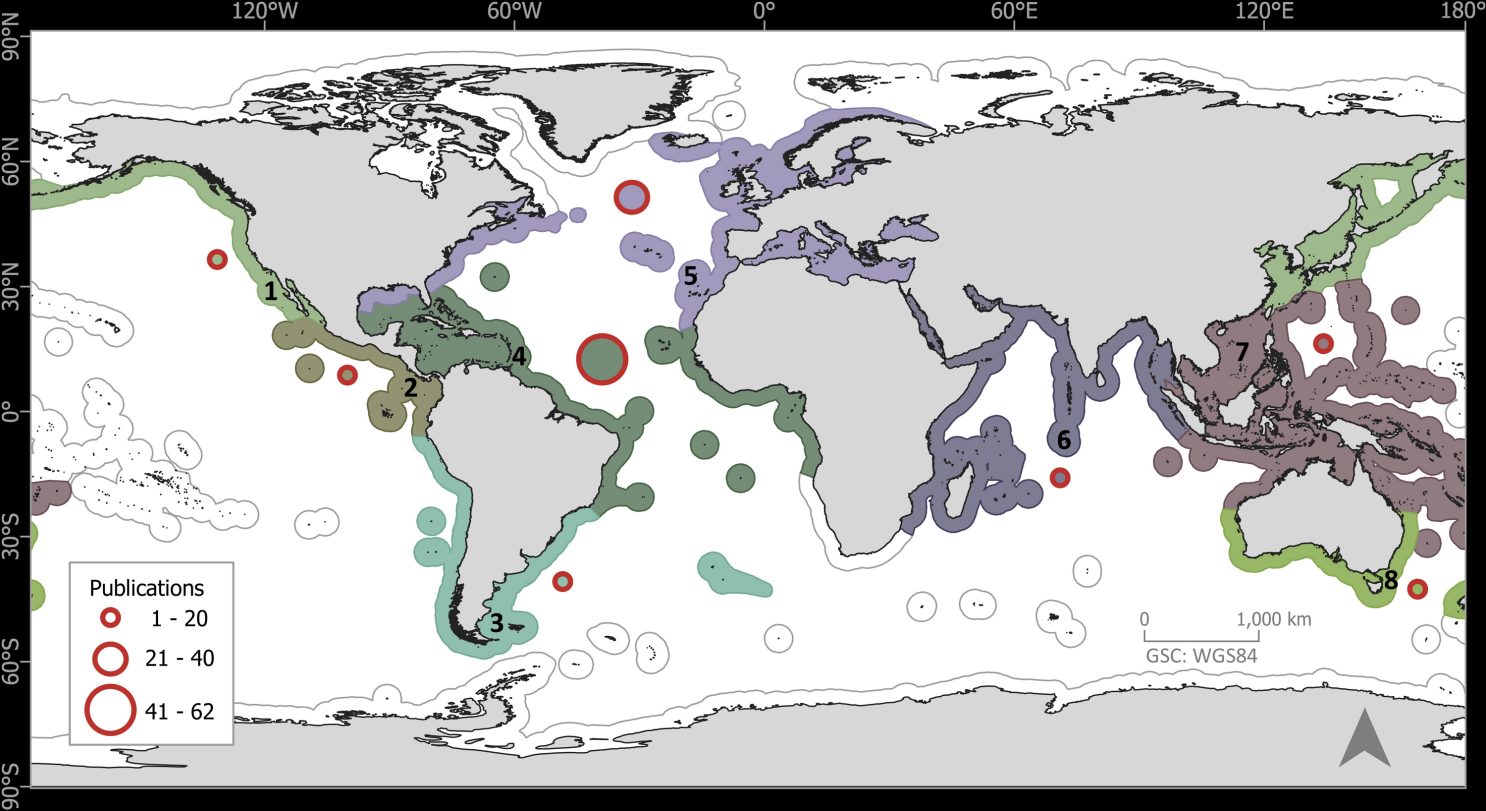
Global distribution of publications with records of epibiotic hydrozoans on *Sargassum*, by marine realm. (1) Temperate Northern Pacific, (2) Tropical Eastern Pacific, (3) Temperate South America, (4) Tropical Atlantic, (5) Temperate Northern Atlantic, (6) Western Indo-Pacific, (7) Central Indo-Pacific, and (8) Temperate Australasia.

The 133 publications with records of epibiotic hydrozoans on *Sargassum* spanned 220 years (1802 to 2022), with most publications (five, 3.75%) in years 1991, 2011, and 2013, followed by four publications in 1909, 2006, and 2018 (Fig. 1). Geographically, the Atlantic Ocean had by far the highest number of publications (100, 75.2%), with most of those and most recent data (*e.g.*, 2020), obtained from the Tropical Atlantic (61, 45.9%) and Temperate Northern Atlantic (31, 23.3%) realms (Fig. 2). Records of epibiotic hydrozoans on *Sargassum* were available from 33 countries, published mainly by authors from the United States (25), Brazil (14), and United Kingdom (14).

**Figure 3:**
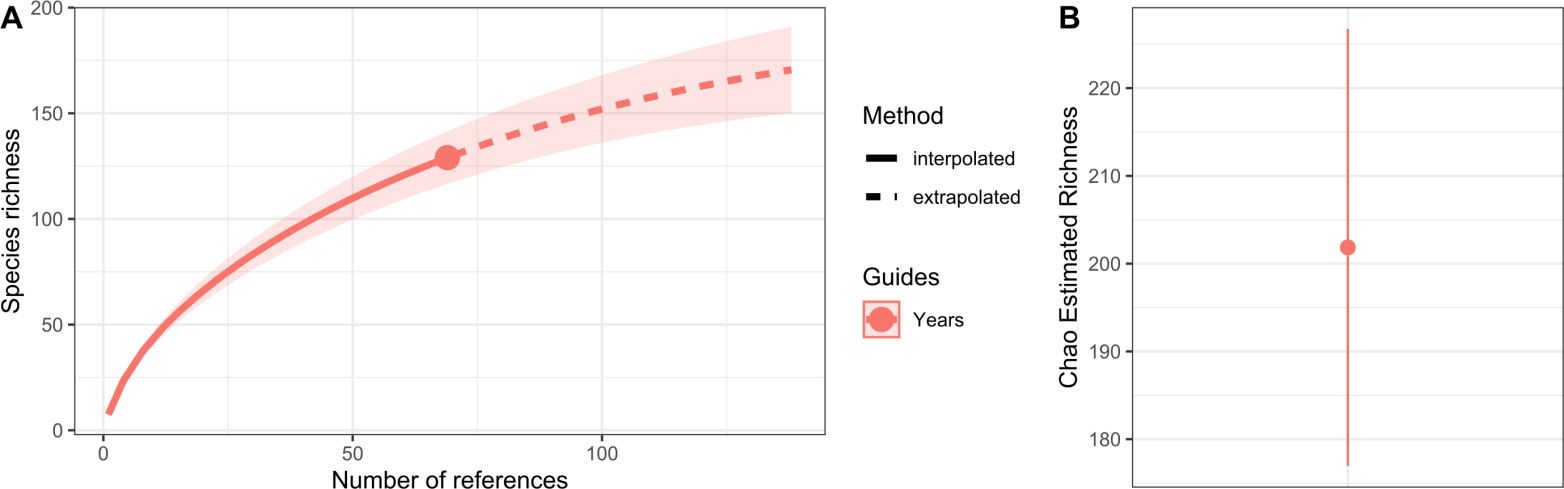
Species richness of hydrozoans in years with available studies with epibiotic hydrozoans on *Sargassum*, in a period from 1802 to 2022. (A) Sample-size-based interpolation and extrapolation curves of species richness with 95% confidence intervals (shaded areas). Symbols on chart indicate the maximum number of species observed for each zone. (B) Chao estimated richness (point) and estimated standard errors (line).

**Figure 4:**
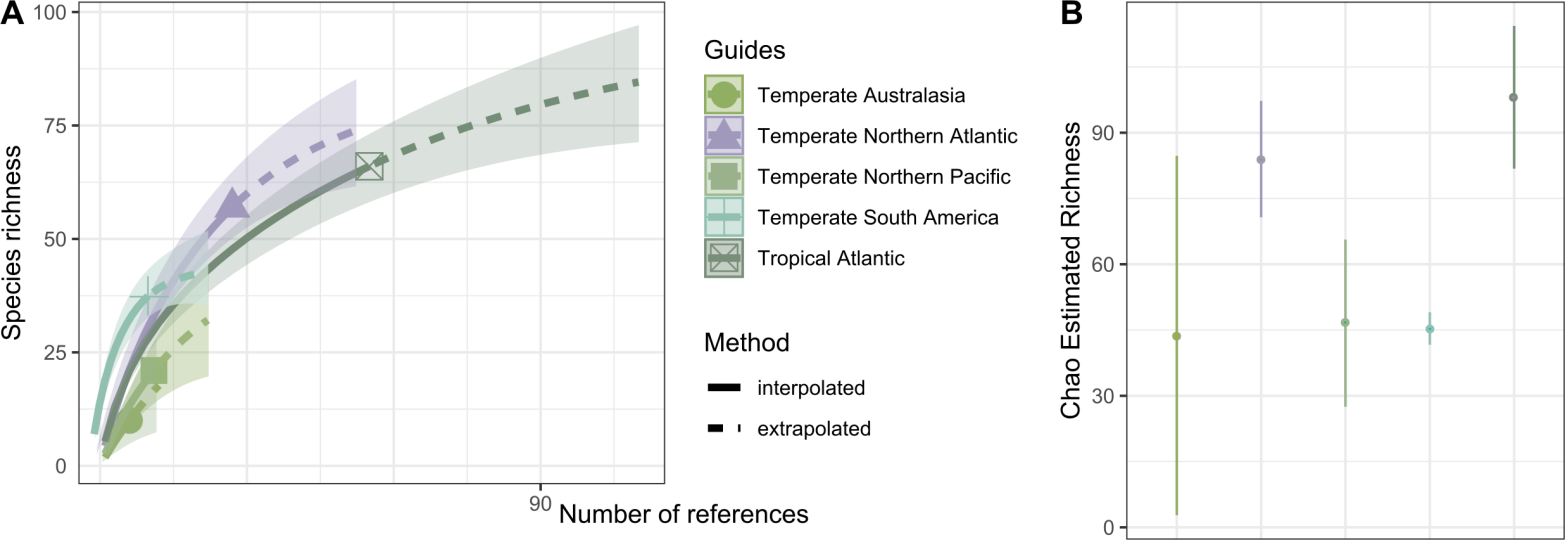
Species richness of hydrozoans in each biogeographic realm. (A) Sample-size-based interpolation and extrapolation curves of species richness with 95% confidence intervals (shaded areas). Symbols on chart indicate the maximum number of species observed for each zone (B) Chao estimated richness (points) and estimated standard errors (lines) for each biogeographic realm.

Hydrozoan richness over time has not yet reached an asymptote (Fig. 3A), and statistical estimates suggest that only about 70% of epibiotic hydrozoan species have been described (Fig. 3B). Species records (104 species) from the Atlantic were included in most of the publications (87), where the greatest numbers of hydrozoan species (64.4%) and publications (64.4%) were recorded from the Tropical Atlantic (Fig. 4A). Temperate Australasia, Temperate Northern Pacific, and Temperate South America, have similarly low values of Chao-estimated species richness relative to the Temperate Northern Atlantic and Tropical Atlantic. However, a higher variation in richness has been observed in the latter two realms (Fig. 4B). Hydrozoans were collected from macroalgae growing over a wide variety of conditions, from water ranging in temperature from 9.0 to 29.6 °C, from the sea surface to 5,100 m in depth (on sunken macroalgae), and at salinities between 29 and 37 PSU.

We compiled 1,128 records of epibiont hydrozoans growing on 26 different species of *Sargassum*, where *Sargassum horridum* and *S. lapazeanum* were the first to be reported as basibionts of hydrozoan species. Two of the *Sargassum* species were holopelagic (*S. fluitans* (Børgesen) Børgesen 1914 and *S. natans* (Linnaeus) Gaillon 1828), and the remaining 24 were benthic. Several different morphotypes were mentioned in the examined literature for holopelagic *Sargassum* species (*Sargassum* morphotypes).

**Figure 5:**
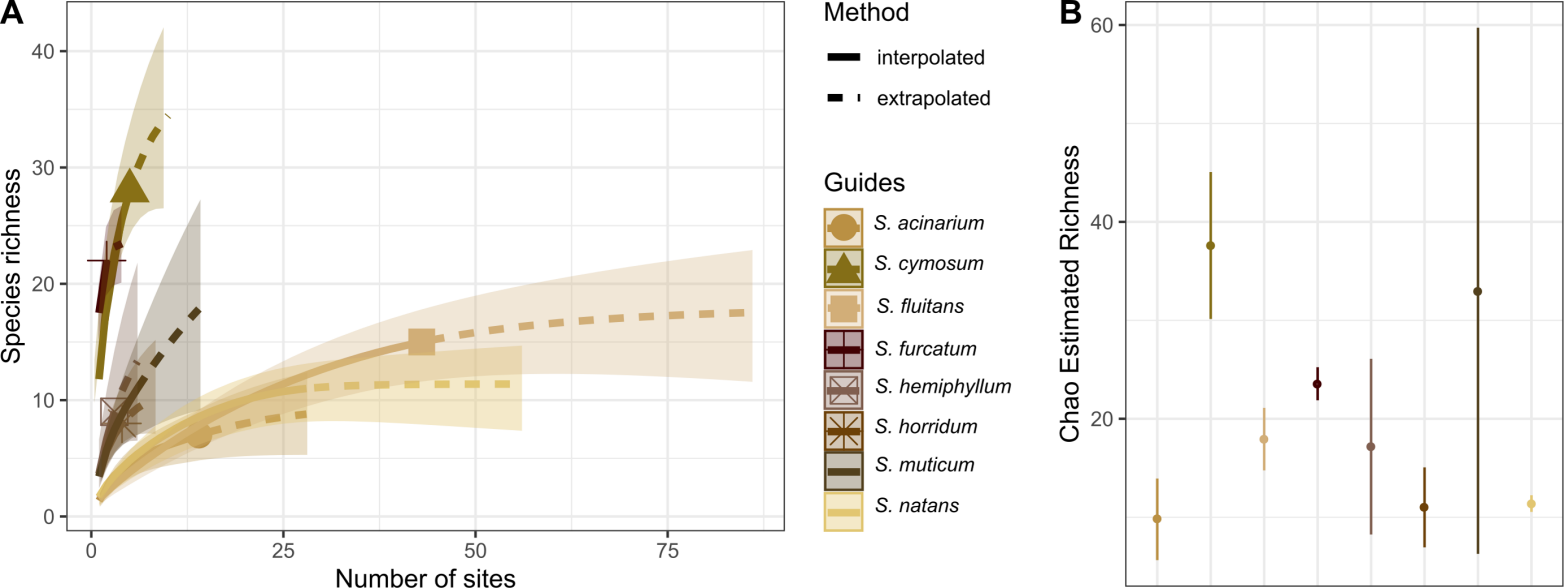
Species richness of hydrozoans on *Sargassum* species. (A) Sample-size-based interpolation and extrapolation curves of species richness with 95% confidence intervals (shaded areas). Symbols on chart indicate the maximum number of species observed for each zone. (B) Chao estimated richness (points) and estimated standard errors (lines) for each species of *Sargassum* indicated.

The holopelagic *S. fluitans* and *S. natans* were the most geographically widespread basibiont species of *Sargassum* sampled (>20 sampling sites), serving as substrates for 14 and 15 hydrozoan species, respectively. In contrast, the benthic species *S. cymosum* C. Agardh 1820 and *S. furcatum* Kützing 1843 were the least geographically widespread species sampled (<10 sites) but showed the highest richness of associated hydrozoans, with 28 and 22 species, respectively (Fig. 5A). In fact, *S. cymosum* has the highest number of hydrozoan species; however, the variation in hydrozoan richness was large (estimated by standard error), especially relative to other benthic basibiont species of *Sargassum* (Fig. 5B). In contrast, the species *S. acinarium* (Linnaeus) Setchell 1933 exhibited the lowest number of associated hydrozoan species (7 spp., Fig. 5B).

**Figure 6:**
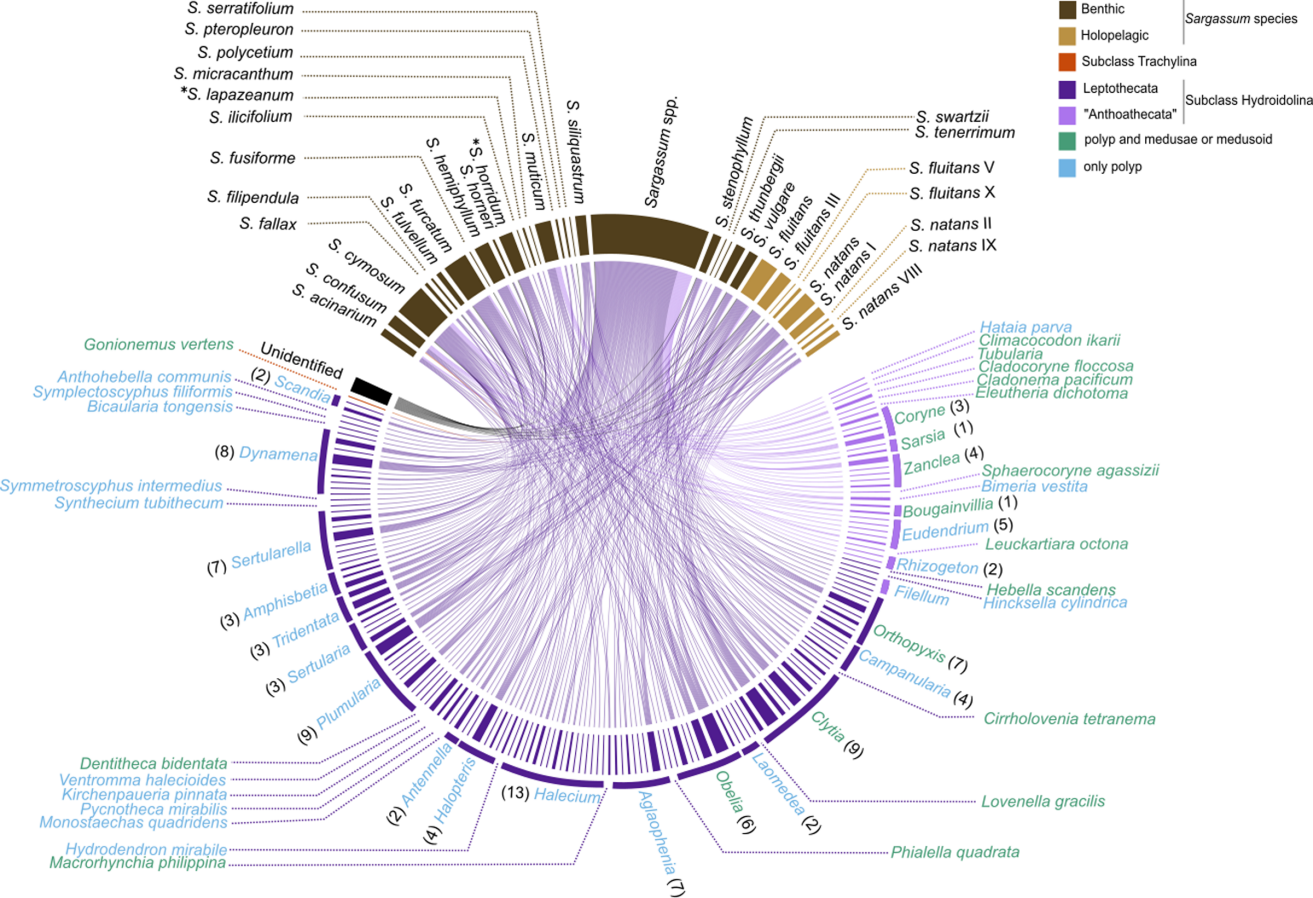
Chord diagram showing the association between hydrozoan and *Sargassum* species obtained from surveyed records of the literature and recent data collected. Entries not identified to species and morphotypes were considered in the analysis. The nodes represent the epibiont hydrozoans on *Sargassum* species (and their morphotypes), linked by lines whose thicknesses represent the number of ecological interactions. Brackets indicate the number of species for each genus of Hydrozoa. An asterisk (*) indicates new data obtained by author.

The survey records of hydrozoan epibionts and *Sargassum* basibionts, including their taxonomic classification and life cycles, show that most hydrozoan species recorded on *Sargassum* belonged to the subclass Hydroidolina (130/131 species), with only one record of a trachyline medusa species (*Gonionemus vertens*, subclass Trachylinae). Among Hydroidolina, most of the records comprised the leptothecate hydrozoan species (superorder Leptothecata, 106 spp.) and anthoathecate hydrozoan (superorder “Anthoathecata”, 24 spp.) (Fig. 6). The leptothecate *Sertularella ampullacea* Fraser, 1938 is a novel epibiotic hydroid on *Sargassum* species.

**Figure 7:**
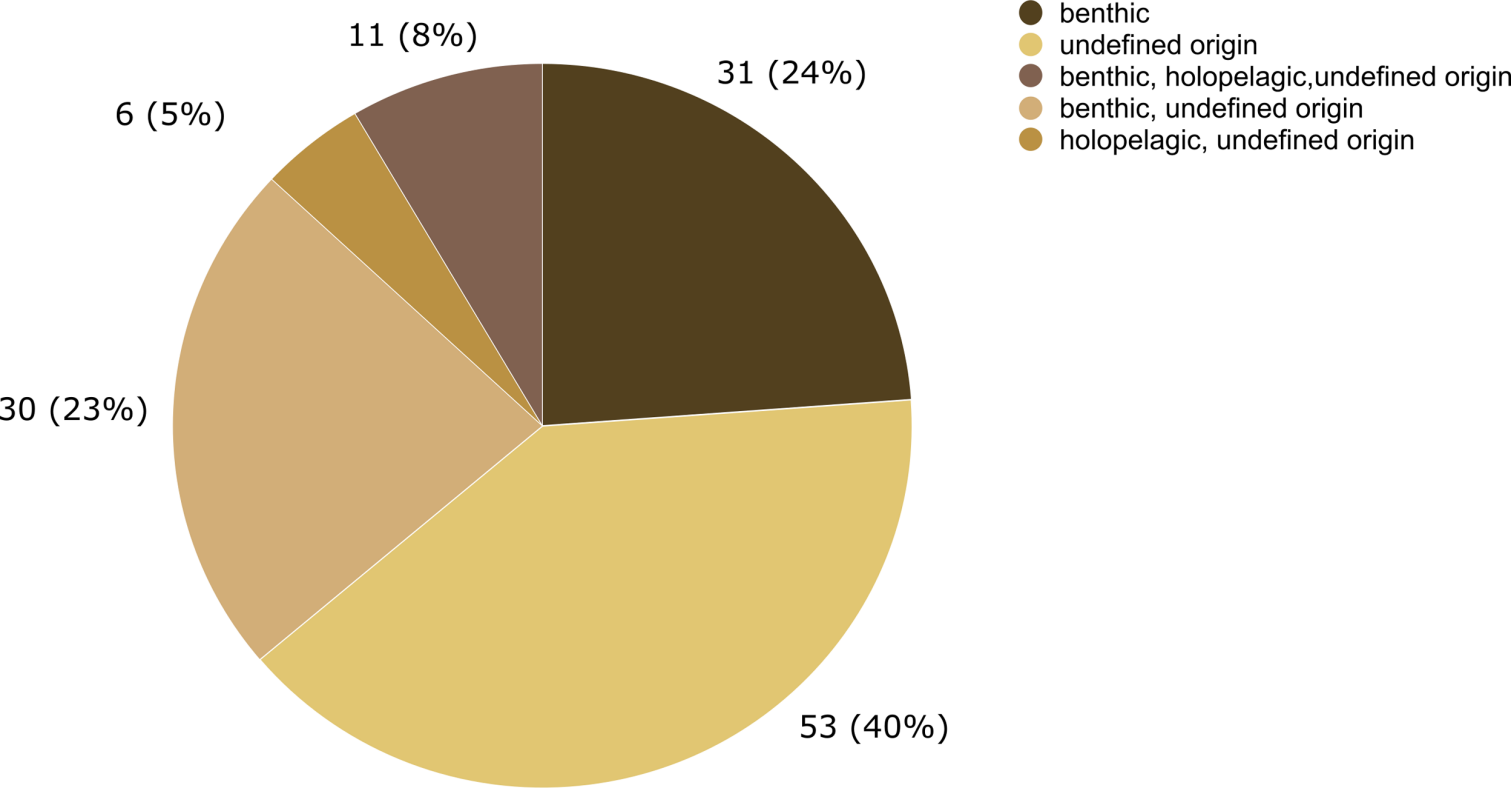
Number of hydrozoan species recorded only on benthic or holopelagic species of *Sargassum* and on *Sargassum* of undefined origin (*i.e.,* information not available in original publications).

The epibiotic leptothecate hydrozoan of *Sargassum* comprised three orders, Macrocolonia (69 spp.), Statocysta (32 spp.), and Lafoeida (one species) and four other species belonging to the family Hebellidae (considered *incertae sedis* in Maronna et al., 2016). Among Macrocolonia, the majority of the species belonged to the most speciose families: Haleciidae (14 spp.), Sertulariidae (17 spp.), and Plumulariidae (10 spp.). Similarly, the genera *Halecium*, *Clytia*, and *Plumularia* showed the highest number of epibiotic species of hydrozoans, comprising 13, nine and nine species, respectively. Among the anthoathecate hydrozoans, 12 species belonged to the order Capitata, 10 to the paraphyletic order Filifera, and two to the order Aplanulata (Fig. 6). The family Eudendriidae (Filifera) comprised the highest number of recorded species (five), all *Eudendrium* spp. The leptothecate species *Obelia dichotoma* (Linnaeus, 1758) and *O. geniculata* (Linnaeus, 1958) were attached to the most species of *Sargassum* (nine and eight *Sargassum* species, respectively) followed by *Clytia hemisphaerica* (Linnaeus, 1767), *Plumularia strictocarpa* Pictet, 1893, and *Sertularella miurensis* Stechow, 1921 (seven different species of *Sargassum* each) (Fig. 6). Thirty-one hydrozoan species have only been reported on benthic species of *Sargassum,* and no hydrozoan species have only been found on holopelagic *Sargassum* (Fig. 7).

**Figure 8:**
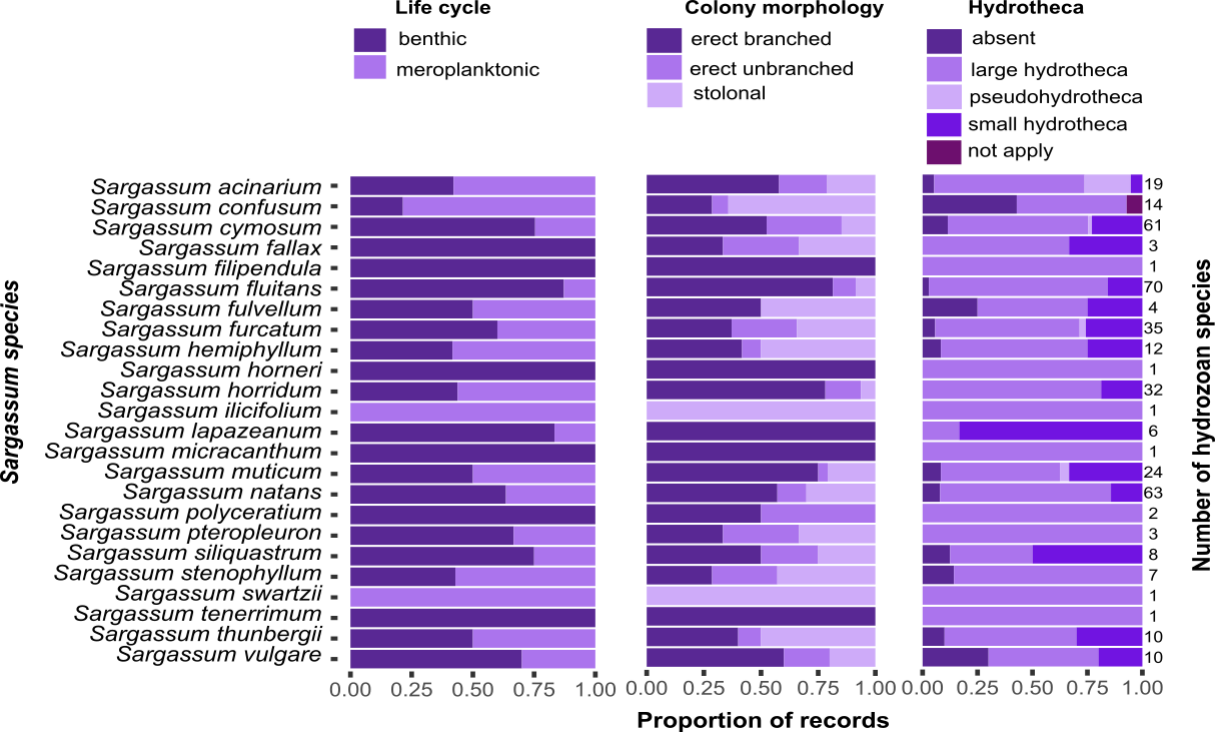
Proportions of the life cycle, colony morphology, and hydrotheca type for hydrozoans recorded growing on each *Sargassum* species studied. Total number of hydrozoans recorded for each *Sargassum* species is shown on the right margin of the charts.

Most of the hydrozoan species growing on *Sargassum* were benthic (*i.e.,* without a medusa/medusoid stage, 92 spp.), had erect, branched colonies (68 spp.), or had hydrothecae (106 spp.) (Fig. 8). However, there was no clear pattern between the composition of hydrozoan species and *Sargassum* species relative to *Sargassum* traits (NMDS, Stress 0.19; PERMANOVA, *p* > 0.6) (Fig. S1, Tables S2 and S3).

## Discussion

Although records of epibiotic hydrozoans on *Sargassum* date to the beginning of the 19th century (Bosc, 1802), it was not until the mid-20th century that the number of studies increased substantially. *Sargassum* species are widely distributed throughout tropical and temperate oceans (Coston-Clements et al., 1991; Yip, Randolph & Danwei, 2020). However, most studies and records of epibiotic hydrozoans are from the Tropical Atlantic and Temperate Northern Atlantic realms, particularly from the east coast of the United States and the Sargasso Sea. This epibiotic species apportionment is consistent with the known distribution of holopelagic *Sargassum* species and morphotypes, which have been extensively studied in these two realms (*e.g.*, Ryland, 1974; Niermann, 1986; Calder, 2013; Govindarajan et al., 2019). Although there has been an increase over time in the number of studies reporting hydrozoans growing on *Sargassum* (with a maximum of five per year), many *Sargassum* species still need to be studied as potential basibionts. Indeed, only 26 of more than 300 accepted species of *Sargassum* (Guiry & Guiry, 2022) have been reported as basibiont epibiotic hydrozoans.

This study identified 92 epibiotic hydrozoan species in addition to the 39 species previously recorded growing on *Sargassum* (cf. Oliveira & Marques, 2007). This number of species is certainly an underestimate; species richness should increase with more sampling, especially if the understudied marine realms are examined more. Furthermore, we found few specific hydrozoan species growing on *Sargassum* species, which corroborates the general view that most hydroids are generalists in their use of substrates (*i.e.,* commonly colonizing macrophytes, other invertebrates, floating objects, and rocks) (Congdon, 1907; Ritchie, 1909; Fraser, 1912; Stechow, 1912).

In this study, 31 hydrozoan species were reported only on benthic S*argassum,* in contrast, zero species were reported only on holopelagic *Sargassum*. This difference in basibiont preference is probably due to the opportunistic nature of many macrofaunal species associated with macrophytes (Vandendriessche et al., 2006). In addition, epifauna dispersal is constrained by the swimming ability of distinct life stages of species and their relationship to the surrounding community. For example, competitive and predatory interactions and substrate availability (Gutow et al., 2015) are modulated by biological and physical processes acting at various spatial scales, from local to global (Bradbury & Snelgrove, 2001). Therefore, the likelihood that a benthic hydrozoan settles on a benthic *Sargassum* is evidently higher than its likelihood of settling on a holopelagic *Sargassum*, whose populations disperse by drifting.

Nine publications recorded the presence of epibiotic hydroids on different portions or structures of sargasso thalli (Leloup, 1932; Ryland, 1974; Niermann, 1986; Bandel & Wedler, 1987; Varela, Ortiz & Lalana, 2005; Abé et al., 2013; Mendoza-Becerril et al., 2020). Species richness of epibiotic invertebrates varies across algal thalli (Ryland, 1974; Mendoza-Becerril et al., 2020), and they can have different rates of larval settlement and metamorphosis depending on the type of algae and depth (Ritson-Williams et al., 2014). However, differences in algal architectures can directly affect habitat complexity by decreasing or increasing the substrate area available (*i.e.,* by affecting the area available for colonization and refuge) (Chemello & Milazzo, 2002).

In this study, the higher species richness observed in leptothecate species growing on *Sargassum* is consistent with the great disparity in the number of epibiont species of the superorders “Anthoathecata” and Leptothecata reported on *Sargassum* in previous studies (cf. Mendoza-Becerril et al., 2020). This difference may be a consequence of the disparity in the species richness observed for these two groups globally (1,285 and 2,137 spp., respectively) (WoRMS, 2022). However, other factors may contribute to the observed disparity in species richness, such as morphological differences in the presence of a chitinous exoskeleton covering the hydranths (in hydrotheca) and gonophores (in gonotheca) in Leptothecata. Perisarc is known to protect soft tissues from mechanical damage and may be a particularly important protective layer among sessile epibiotic species in habitats with medium to high hydrodynamic energies where the potential for abrasion is high (Riedl, 1966; Gili, Murillo & Ros (1989); Marques et al., 2007; Mendoza-Becerril et al., 2020).

However, most species reported in the reviewed studies possessed a hydrorhiza, which allows a hydroid colony to attach to a variety of substrates and provides it with a resting stage to weather unfavorable conditions (Mills et al., 2007; Jaubet & Genzano, 2011). Moreover, sessile epibionts with a metamorphosis stage in their life cycles typically settle at the larval stage during favorable conditions and on available substrates, such as macroalgae (Hadfield, 1986; Sommer, 1992), by producing adhesive granules, sticky mucus (Stricker, 1989; Sommer, 1992), or growing their stolons (Cerrano et al., 2001). Some authors (*e.g.*, Song et al., 2019), believe that hydroids are able to expand their geographical ranges by settling on macroalgae that passively drift with currents, similar to drifting debris (Choong et al., 2011; Calder et al., 2014). In addition to horizontal floating dispersal, macroalgae also vertically disperse hydroids when they sink, as demonstrated by records of hydroid species attached to thalli of sunken *Sargassum* recorded from deep waters (2,810 m, 3,550 m, and 5,100 m) (Fraser, 1947; Calder, 1996).

Most records of hydrozoans on *Sargassum* are comprised of hydrozoan species with benthic life cycles (*i.e.,* without a medusa stage), mainly hydrozoans in the order Macrocolonia (Leclère et al., 2009; Maronna et al., 2016). Such species usually exhibit perennial colonies, although some species may become dormant under seasonally unfavorable (usually colder) conditions (*e.g.*, some aglaopheniids) (Di Camillo et al., 2017). Seasonal cycles of activity, in which colonies reproduce asexually or disperse, are important strategies that facilitate the colonization of algal substrates (*e.g.*, *Halecium* sp., cf. Gravier-Bonnet & Bourmaud, 2005). Furthermore, most records of Macrocolonia comprise species with erect and branched colonies (Fig. 8), which are considered derived traits and are usually associated with the presence of fixed gonophores (Leclère et al., 2009). In contrast, other hydrozoans with hydromedusae in their life cycles often possess tentacled adhesive structures that help attach them to a substrate (*e.g.*, hydrozoans populating coastal marine vegetation) (Tambs-Lyche, 1964; Gulliksen, 1971; Bakker, 1980). Such adhesive structures occur in the trachymedusan, *Gonionemus vertens* (Russell, 1953; Gulliksen, 1971; Honegger, 1984), and in other genera, including *Aglauropsis, Cladonema, Cubaia, Eleutheria, Gonionemus, Olindias, Solionema,* and *Vallentinia* (Bakker, 1980).

However, we found no specific pattern between *Sargassum* species and hydrozoan traits. Additional studies will be necessary with other species of *Sargassum* to determine possible patterns between algal structure and epibiont composition.

## Conclusions

A total of 131 hydrozoan species was recorded on 26 species of *Sargassum* over the years 1802 to 2022, with the highest number of hydrozoan species recorded on *S. cymosum* (28 spp.) and the smallest number on *S. acinarium* (seven spp.). The number of studies examining epibiotic hydrozoans on *Sargassum* has increased since the mid-20th century, and most studies and records originated from the Tropical Atlantic and Temperate Northern Atlantic, particularly from the east coast of the United States and the Sargasso Sea. However, the highest species richness of epibiotic hydrozoans has been recorded from the Tropical Atlantic (67 spp.). Based on our review, epibiont hydrozoan species richness increased by 70%, and more sampling of *Sargassum* for hydrozoan species, especially among benthic *Sargassum,* would likely help reveal potential patterns between sargasso and hydrozoan epibionts.

##  Supplemental Information

10.7717/peerj.15423/supp-1 Supplemental Information 1 Supplementary tables and figure

## Additional Information and Declarations

### Data Availability

The following information was supplied regarding data availability: Carral-Murrieta, Cecilia Odette, Estrada-González, Mariae, Rosales Catalán, Leonela, León Cisneros, Karla, Mazariegos Villareal, Alejandra, Serviere Zaragoza, Elisa, & Mendoza-Becerril, María A. (2023). A review of epibiont hydrozoans on Sargassum [Data set]. Zenodo. https://doi.org/10.5281/zenodo.6971278.
