# Supplementary material for: A survey of epibiont hydrozoans on Sargassum
Source: PeerJ. 2023 May 30;11:e15423. doi: 10.7717/peerj.15423 (PMC10237180; doi:10.7717/peerj.15423)
Supplement: Supplemental Information 1 [file peerj-11-15423-s001.docx]

**Table S1:** Collection sites of *Sargassum* in La Paz Bay, Baja California Sur (BCS), Mexico.

| *Sargassum* species | Site | Date | Number of thalli |
| --- | --- | --- | --- |
| *Sargassum horridum* Setchell & N.L. Gardner | San Juan de la Costa | March 2018 | 1 |
|  |  | July 2021 | 1 |
|  | El Sauzoso | May 2018 | 1 |
|  | Agua de Yepiz | March 2021 | 1 |
|  | El Califin | May 2018 | 3 |
|  | El Cajete | July 2021 | 1 |
|  | Playa Caimancito | April 2018 | 17 |
|  |  | August 2018 | 1 |
| *Sargassum lapazeanum* Setchell & N.L. Gardner | San Juan de la Costa | March 2018 | 3 |
|  |  | July 2021 | 1 |

**Table S2:** PERMANOVA results for each *Sargassum* trait (habit and region) using Bray-Curtis dissimilarity. df: degrees of freedom; SS: square sum, MS: mean sum of squares, *F* Model: *F* value by permutation, *statistical significance with *p*<0.05 based on 999 permutations.

| Source | df | SS | MS | *F* model | R^2^ | Pr(>*F*) |
| --- | --- | --- | --- | --- | --- | --- |
| **Habit** | 2 | 1.113 | 0.556 | 1.3694 | 0.110 | **0.04*** |
| Residuals | 22 | 8.943 | 0.406 |  | 0.889 |  |
| Total | 24 | 10.056 |  |  | 1.000 |  |
| **Region** | 6 | 4.557 | 0.759 | 2.486 | 0.453 | **0.01*** |
| Residuals | 18 | 5.499 | 0.305 |  | 0.546 |  |
| Total | 24 | 10.056 |  |  | 1.000 |  |

**Table S3:** Pairwise adonis test between groups of every *Sargassum* trait (habit andregion). df: degrees of freedom; SS: square sum, *F* Model: *F* value by permutation, *statistical significance with *p*<0.05. Adjusted *p*: Bonferroni adjustment. TNA: Temperate Northern Atlantic, TNP: Temperate Northern Pacific, TAS: Temperate Australasia, TA: Tropical Atlantic, TSA: Temperate South America, WIP: Western Indo-Pacific.

| **Habit** | | | | | | |
| --- | --- | --- | --- | --- | --- | --- |
| Pairs | df | SS | *F* model | R^2^ | *p* value | Ajusted *p* |
| Benthic vs drifting | 1 | 0.454 | 1.068 | 0.0484 | 0.350 | 1.000 |
| Benthic vs holopelagic | 1 | 0.671 | 1.667 | 0.0735 | 0.027 | 0.081 |
| Drifting vs holopelagic | 1 | 0.482 | 1.951 | 0.493 | 0.333 | 1.000 |
| **Region** | | | | | | |
| TNA vs TNP | 1 | 0.560 | 1.927 | 0.194 | 0.204 | 1.00 |
| TNA vs widespread | 1 | 0.428 | 1.303 | 0.206 | 0.265 | 1.00 |
| TNA vs TAS | 1 | 0.500 | NA | 1.000 | NA | NA |
| TNA vs TA | 1 | 0.537 | 2.191 | 0.522 | 0.250 | 1.00 |
| TNA vs TSA | 1 | 0.409 | 1.103 | 0.524 | 0.666 | 1.00 |
| TNA vs WIP | 1 | 0.583 | 1.750 | 0.466 | 0.500 | 1.00 |
| TNP vs widespread | 1 | 1.084 | 3.549 | 0.214 | 0.008 | 0.16 |
| TNP vs TAS | 1 | 0.667 | 2.294 | 0.222 | 0.098 | 1.00 |
| TNP vs TA | 1 | 1.275 | 4.526 | 0.311 | 0.001 | 0.02 |
| TNP vs TSA | 1 | 0.644 | 2.151 | 0.192 | 0.096 | 1.00 |
| TNP vs WIP | 1 | 1.168 | 3.903 | 0.281 | 0.007 | 0.14 |
| Widespread vs TAS | 1 | 0.616 | 1.875 | 0.272 | 0.145 | 1.00 |
| Widespread vs TA | 1 | 0.882 | 2.895 | 0.292 | 0.011 | 0.22 |
| Widespread vs TSA | 1 | 0.175 | 0.521 | 0.0799 | 0.955 | 1.00 |
| Widespread vs WIP | 1 | 0.886 | 2.686 | 0.277 | 0.011 | 0.22 |
| TAS vs TA | 1 | 0.627 | 2.558 | 0.561 | 0.250 | 1.00 |
| TAS vs TSA | 1 | 0.542 | 1.460 | 0.593 | 0.333 | 1.00 |
| TAS vs WIP | 1 | 0.921 | 1.750 | 0.466 | 0.500 | 1.00 |
| TA vs TSA | 1 | 0.479 | 1.669 | 0.357 | 0.300 | 1.00 |
| TA vs WIP | 1 | 0.921 | 3.185 | 0.443 | 0.100 | 1.00 |
| TSA vs WIP | 1 | 0.643 | 1.860 | 0.382 | 0.200 | 1.00 |


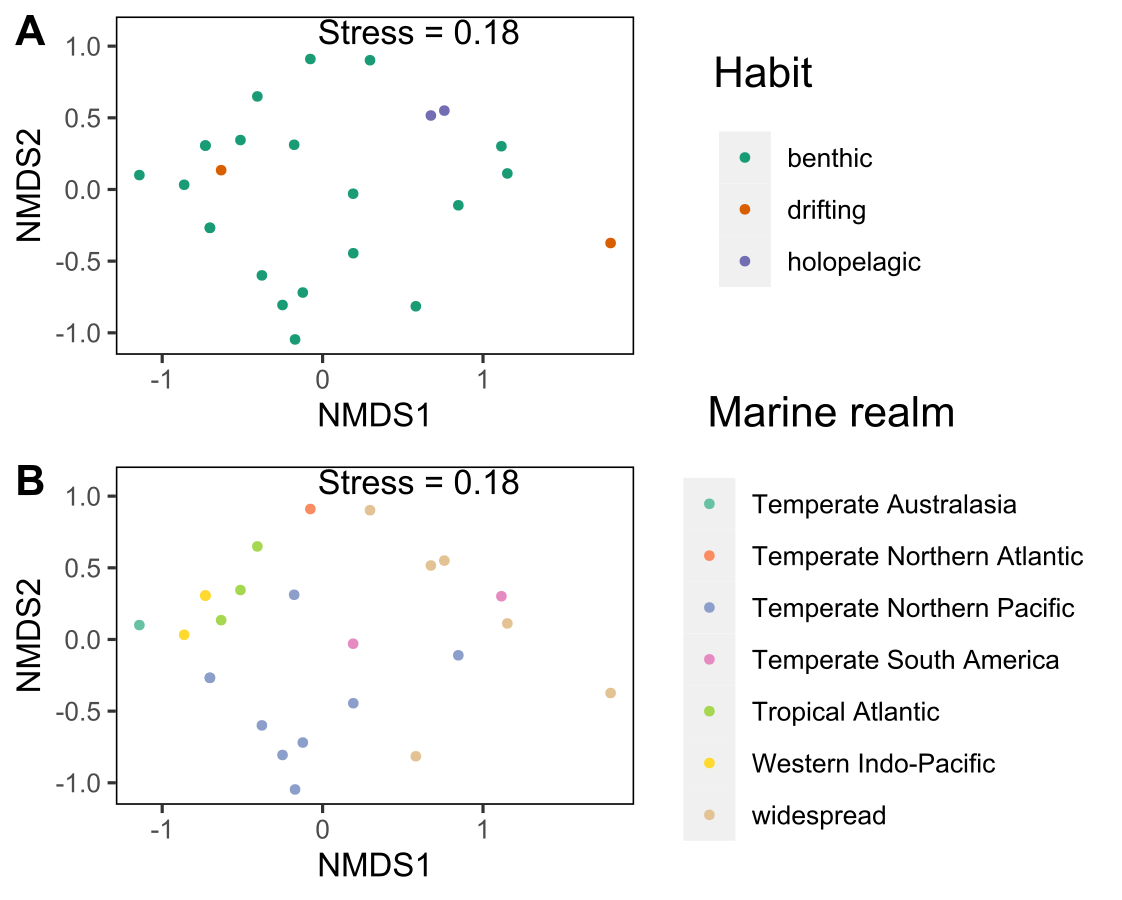


**Figure S1:** Non-metric multidimensional scaling (NMDS) ordination of the composition of hydrozoan species between *Sargassum* species colored by *Sargassum* habit (A) and (B) geographic realm.
